# The Relationship Between Evaluative Concerns Perfectionism and Depression in Early Adults: The Mediating Effect of Rumination and the Mediated Moderation Effect of Mindfulness

**DOI:** 10.3390/bs15121692

**Published:** 2025-12-06

**Authors:** Hyun-Ju Chae, Sung Man Bae

**Affiliations:** 1 Department of Psychology, Graduate School, Dankook University, Cheonan 31116, Republic of Korea; chj980122@naver.com; 2 Department of Psychology and Psychotherapy, Dankook University, Cheonan 31116, Republic of Korea

**Keywords:** evaluative concerns perfectionism, rumination, mindfulness, depression, mediated moderating effect

## Abstract

This study examined the mediating and moderating effect of mindfulness through rumination on the relationship between evaluative concerns perfectionism and depression in early adults. For this study, an online self-report survey was conducted on 436 participants aged 19 to 35, and 405 participants (179 men, 226 women) were included in the final analysis. The mediating, moderating, and mediated moderating effects were verified using the PROCESS macro for SPSS v3.5. It was found that evaluative concerns perfectionism had a significant effect on depression, and this relationship was mediated by rumination. As a result, mindfulness (nonjudging of experience and acting with awareness) had a partially mediated moderating effect on the relationship between evaluative concerns perfectionism, rumination, and depression.

## 1. Introduction

Depression refers to a psychological state characterized by persistent sadness, loss of interest or pleasure, decreased energy, and changes in negative thinking that last for a certain period and cause difficulties in daily functioning (Henkel et al., 2009). Depression can cause difficulties in daily life, such as job adjustment, academic performance, and interpersonal relationships (S. M. Kwon, 2013). In particular, people in their 20s and 30s have a higher prevalence of depressive disorder than those in other age groups (Korea Disease Control and Prevention Agency, 2024). As a result, it is necessary to prevent, detect early, and intervene in depression in early adulthood.

An increase in depression in Korean youth is related to a competitive social atmosphere, a slowly growing unemployment rate, and an uncertain future (J. Kim et al., 2018). In particular, the competitive social atmosphere requires individuals to have better abilities and capacities (J. M. Kim, 2009). This social mood can lead individuals to set excessively high standards and force them to perform more, thereby reinforcing their propensity to pursue perfection (I. J. Kwon, 2010). In particular, Korean youth often encounter situations where they are evaluated by others and thus saturated with ideas of perfectionism (J. W. Choi, 2021).

Perfectionism can be defined as setting excessively high standards and evaluating oneself strictly according to them (Frost et al., 1990). It is not a single-dimensional concept with maladaptive characteristics but a multidimensional concept that includes both adaptive and maladaptive elements (Dunkley et al., 2003). It can be classified into two dimensions: personal standards perfectionism, corresponding to adaptive perfectionism, and evaluative concerns perfectionism, corresponding to maladaptive perfectionism (Dunkley et al., 2000). Personal standards perfectionism is generally considered desirable because it involves setting high standards to motivate oneself and improve performance, whereas evaluative concerns perfectionism is considered undesirable as it involves excessive worry about others’ evaluation and fear of making mistakes, which can increase vulnerability to negative emotions and depression (Dunkley et al., 2000; H. H. Kim & Kim, 2011; J. H. Choi, 2020). Individuals with a high level of evaluative concerns perfectionism easily experience self-criticism and despair when they fail to achieve their goals, and they are found to be vulnerable to depression (Powers et al., 2004). Overgeneralization, dichotomous thinking, and self-defeating thinking, which are maladaptive and distorted cognitive characteristics of evaluative concerns perfectionism, can lead to depression (Shafran et al., 2002). In fact, a longitudinal study has also shown that evaluative concerns perfectionism significantly predicts depression (Sherry et al., 2014).

Numerous previous studies have focused on rumination, a cognitive variable, as a factor to explain the relationship between maladaptive perfectionism and depression. Rumination can be defined as thoughts and actions that repeatedly address negative emotions and their implications (Nolen-Hoeksema & Morrow, 1991). In particular, maladaptive perfectionists ruminate more than adaptive perfectionists and non-perfectionists (H. J. Kim & Son, 2006). Rumination is closely related to depression as well as perfectionism. Several previous studies also found that rumination has a positive effect on depression and prolongs its duration (Nolen-Hoeksema, 2000; Yi, 2008).

Based on previous studies, it is possible to assume a relationship between perfectionism, rumination, and depression. Harris et al. (2008) confirmed the mediating effect of rumination on the relationship between maladaptive perfectionism and depression. J. H. Choi (2020) stated that rumination mediates the relationship between evaluative concerns perfectionism and depression and is a factor that compounds negative thoughts of evaluative concerns perfectionism and worsens depression. Therefore, for therapeutic intervention and the prevention of depression caused by perfectionism, it is necessary to explore factors that can buffer the relationship between perfectionism, rumination, and depression.

Previous studies have suggested that the focus of intervention against perfectionists should be on the acceptance attitude (Lundh, 2004). Acceptance is an important element of mindfulness and is defined as intentional and receptive attention to current thoughts, sensations, and emotions (Kabat-Zinn, 1994). Several studies have shown that the effect of rumination on psychological health varies depending on the mindfulness level (Hong, 2016; Park & Sung, 2008; Shahar & Nitzan-Assayag, 2020). A recent study also showed that mindfulness has a moderating effect on the relationship between rumination and depression (Yoo et al., 2020). These results suggest that if the level of mindfulness is high, the possibility of negative emotions such as depression is reduced, even if the tendency to ruminate negatively about the situation is prominent. A study on the effectiveness of programs on perfectionism and mindfulness showed that mindfulness is effective at reducing maladaptive perfectionism and its negative effects.

Previous studies have confirmed that mindfulness alleviates perfectionism, rumination, and depression; however, few studies have examined the relationship between the subfactors of mindfulness and depression. Mindfulness comprises five factors: nonreactivity, observing, acting with awareness, describing, and nonjudging of experience (Baer et al., 2006). Nonjudging of experience means to avoid making judgments such as good/bad, right/wrong, or worthy/unworthy. Nonreactive individuals are not overwhelmed by inner experience and do not respond immediately. Observing refers to paying attention to and observing internal and external stimuli. Acting with awareness refers to fully engaging in one’s current activities without being distracted. Description refers to verbally describing and naming observed phenomena (D. R. Won, 2007).

In summary, studies on the mediating and moderating effects explaining the relationship between perfectionism and depression are being conducted individually, but efforts to integrate them are lacking. Therefore, this study aimed to comprehensively examine the relationship between evaluative concerns perfectionism, rumination, depression, and mindfulness through an analysis of mediated moderating effects and to verify the role of mindfulness in detail. In addition, existing studies have limitations in that, although mindfulness is a multidimensional concept, they mainly analyzed it using the total score of the mindfulness scale (Baer et al., 2006). Therefore, in this study, mindfulness was divided into five subfactors to examine its moderating effect.

## 2. Methods

### 2.1. Participants

This study conducted a survey targeting adults aged between 19 and 35 years residing in the Republic of Korea; this was conducted as a self-reported questionnaire using Google Survey. Among the 436 participants, data from 405 were included in the analysis. Responses from 31 participants were excluded because they appeared not to have engaged meaningfully with the survey, which was determined based on patterns of inattentive responding, such as selecting the same answer across all items or providing logically inconsistent responses. The sample included 179 (44.2%) men and 226 (55.8%) women with an average of 27.15 years and a standard deviation of 4.76. All research procedures, including data collection, were approved by the Institutional Review Board of Dankook University (approval number: 2022-06-005-001).

### 2.2. Measures

#### 2.2.1. Evaluative Concerns Perfectionism

The Evaluative Concerns Perfectionism Scale consists of a combination of socially prescribed perfectionism, a subfactor of the Hewitt Multidimensional Perfectionism Scale (HMPS) developed by Hewitt and Flett (1991), and doubt about performance and concern about mistakes, a subfactor of the Frost Multidimensional Perfectionism Scale (FMPS) developed by Frost et al. (1990). In this study, the Evaluative Concerns Perfectionism Scale consisted of doubts about performance (four items), concern about mistakes (nine items), and socially prescribed perfectionism (15 items). The scale consisted of 28 items, with performance and concern about mistakes rated on a 5-point Likert scale (1 = not at all true, 5 = completely true). Socially prescribed perfectionism was rated on a 7-point Likert scale (1 = not at all true, 7 = completely true). Higher total scores were associated with a higher degree of perfectionism. The Cronbach’s alpha for the data in this study was 0.80. By subfactor, concern about mistakes was 0.83, doubt about performance was 0.77, and socially prescribed perfectionism was 0.80. Exploratory factor analysis was conducted to identify the validity of the scale structure. First, based on the Kaiser–Meyer–Olkin (KMO) value (0.914) and Bartlett’s test of sphericity (X^2^ = 5116.530, *p* < 0.001), we confirmed that the data were suitable for factor analysis. Factor analysis was performed using the principal axis factoring extraction method and oblique rotation (direct oblimin) without specifying the number of factors. The results showed three factors with eigenvalues greater than 1. Considering the scree plot and the pattern and structure matrices comprehensively, a three-factor structure was found to be appropriate. These results suggest that the three substructures (doubts about performance, concern about mistakes, and socially prescribed perfectionism) constructed to measure evaluative concerns perfectionism in this study are valid.

#### 2.2.2. Rumination

The Korean version of the Ruminative Response Scale (K-RRS) that had been developed by S. J. Kim et al. (2010) was used to measure rumination, with a specific focus on brooding, a maladaptive and negatively valenced aspect of rumination that is strongly associated with depressive symptoms. The scale consisted of 22 items rated on a 4-point Likert scale (1 = never, 4 = always), with higher total scores indicating greater levels of rumination. In the present study, rumination was conceptualized primarily as brooding. The Cronbach’s alpha for the data in this study was 0.89.

#### 2.2.3. Mindfulness

To measure the mindfulness of respondents, the Korean version of the Five Facets of Mindfulness Questionnaire (K-FFMQ) that had been developed by D. Won and Kim (2006) was used. Originally, this was a 7-point Likert scale; however, Song and Bae (2020) reconstructed it as a 5-point Likert scale (1 = strongly disagree, 5 = strongly agree). Higher total scores were associated with higher levels of mindfulness. This scale consisted of nonjudging of experience (eight items), describing (eight items), acting with awareness (eight items), nonreactivity (seven items), and observing (eight items), for a total of 39 items. The Cronbach’s alpha for the data in this study was 0.82. By subfactor, nonjudging of experience was 0.81, describing was 0.79, acting with awareness was 0.85, nonreactivity was 0.79, and observing was 0.82.

#### 2.2.4. Depression

The Center for Epidemiological Studies Depression Scale (CES-D), developed by Chon (2001), was used to measure respondents’ depressive symptoms. This scale included 20 items about depressive symptoms experienced during the week, rated on a 4-point Likert scale (0 = rarely, 3 = all the time). Higher total scores were associated with more depressive symptoms. The Cronbach’s alpha for the data in this study was 0.92.

#### 2.2.5. Control Variables

Age, sex, and subjective socioeconomic status were assessed and included as control variables in all analyses. Sex was coded as 0 = men and 1 = women.

### 2.3. Analysis

The data collected in this study were analyzed using IBM SPSS Statistics version 23.0 and the PROCESS macro for SPSS version 3.5 developed by A. F. Hayes (2017). First, frequency analysis was conducted using SPSS 23.0 to confirm the demographic characteristics of the study participants, and Cronbach’s α was calculated to confirm the reliability of each measurement tool. Second, descriptive statistical analysis was conducted to determine the standard deviation, mean, skewness, and kurtosis of the major variables. In addition, Pearson’s correlation coefficient was calculated to determine the relationship between the major variables. Third, age, sex and subjective socioeconomic status were used as control variables. Sex was measured by coding 0 for men and 1 for women. Fourth, the direct effect of evaluative concerns perfectionism on depression and the indirect effect of rumination were verified. Multiple regression analysis was used to verify the mediation model according to Baron and Kenny (1986). In addition, the significance of the indirect effects was verified using bootstrapping in the SPSS PROCESS macro (version 3.5) developed by A. F. Hayes (2017). Fifth, the moderating effect of each subfactor of mindfulness on the relationship between evaluative concerns perfectionism, rumination, and depression was verified. To verify the moderating effect, the interaction effect was analyzed using the PROCESS macro 1 model. In addition, the significance of the simple regression line according to each condition value (Mean − 1SD, Mean, and Mean + 1SD) of the moderating variable was verified to examine detailed interactions (Aiken et al., 1991). Sixth, the PROCESS macro model 15 was used to verify the mediated moderating effect of mindfulness on the relationship between evaluative concerns perfectionism, rumination, and depression.

## 3. Results

### 3.1. Descriptive Statistics and Correlation Analysis Between Major Variables

Evaluative concerns perfectionism was positively correlated with rumination and depression and negatively correlated with mindfulness. Rumination was negatively correlated with mindfulness and positively correlated with depression. Mindfulness was negatively correlated with depression. The descriptive statistics and correlations of the main variables are summarized in Table 1.

### 3.2. Mediation Effect

**Figure 1:**
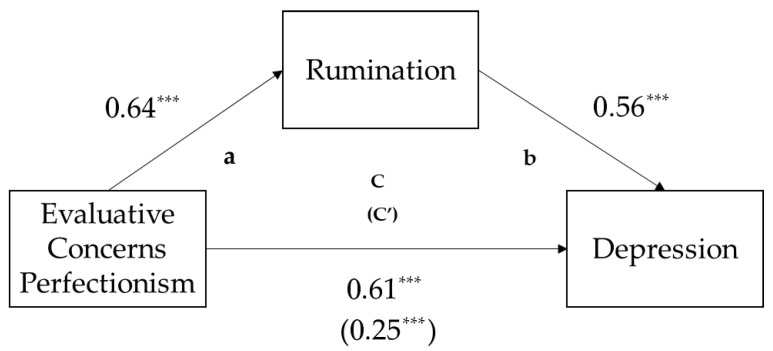
Mediation Model. a: The effect of evaluative concerns perfectionism on rumination; b: The effect of rumination on depression when controlling for evaluative concerns perfectionism; c: The direct effect of evaluative concerns perfectionism on depression; c′: The direct effect of evaluative concerns perfectionism on depression after accounting for the mediator (rumination); *** *p* < 0.001.

The results verifying the mediating effect of rumination on the relationship between evaluative concerns perfectionism and depression are as follows. Refer to the mediation model depicted in Figure 1. In Step 1, the direct effect of the independent variable—evaluative concerns perfectionism—on the dependent variable—depression—was significant (β = 0.61, *p* < 0.001). Next, after confirming the effect of evaluative concerns perfectionism on rumination in Step 2, it was found that evaluative concerns perfectionism significantly predicted rumination (β = 0.64, *p* < 0.001). Finally, in Step 3, rumination had a significant effect on depression (β = 0.56, *p* < 0.001), after controlling for the influence of evaluative concerns perfectionism. At this time, when the influence of rumination was controlled, the effect of evaluative concerns perfectionism on depression decreased compared to the path examined in Step 1 (β = 0.25, *p* < 0.001), but the effect was still significant.

Bootstrapping using SPSS PROCESS macro model 4 was used to more accurately confirm whether the mediating effect verified through the three-step hierarchical multiple regression analysis was statistically significant. The indirect effect coefficient was 0.21, and the lower and upper limits of the 95% confidence interval were 0.17 and 0.25, respectively. In conclusion, the mediating pathway identified in this study was verified.

### 3.3. Moderating Effect

#### 3.3.1. The Moderating Effect of Mindfulness on the Relationship Between Evaluative Concerns Perfectionism and Depression

The results are summarized in Table 2. First, it was found that evaluative concerns about perfectionism (*B* = 0.31, *t* = 11.99, *p* < 0.001) and nonjudgment of experience (B = −0.29, t = −3.29, *p* < 0.01) had a significant effect on depression. In addition, the interaction effect of the two variables (B = −0.02, t = −4.26, *p* < 0.001) was found to have a significant effect on depression, indicating that nonjudging of experience moderated the relationship between evaluative concerns perfectionism and depression. Evaluative concerns perfectionism (B = 0.27, t = 10.97, *p* < 0.001) and acting with awareness (B = −0.54, t = −7.11, *p* < 0.001) had a significant effect on depression. Moreover, the interaction effect of the two variables (B = −0.02, t = −4.87, *p* < 0.001) was found to have a significant effect on depression, indicating that acting with awareness moderates the relationship between evaluative concerns perfectionism and depression. However, describing, observing, and nonreactivity did not moderate the relationship between evaluative concerns perfectionism and depression.

To examine the moderating effect of nonjudging of experience in detail, participants were divided into a high group (Mean + 1SD) and a low group (Mean − 1SD). The group with low nonjudging of experience showed a stronger positive relationship between evaluative concerns perfectionism and depression (B = 0.41, t = 11.98, *p* < 0.001) than the high group (B = 0.22, t = 6.60, *p* < 0.001), supporting the partial moderation effect. Similarly, low acting with awareness strengthened the positive relationship (B = 0.36, t = 11.66, *p* < 0.001) compared to the high group (B = 0.18, t = 5.92, *p* < 0.001).

#### 3.3.2. The Moderating Effect of Mindfulness on the Relationship Between Rumination and Depression

The results are summarized in Table 3. First, rumination (B = 0.57, t = 17.57, *p* < 0.001) had a significant effect on depression, while nonjudging of experience had no significant effect. In addition, the interaction effect of the two variables (B = −0.03, t = −5.05, *p* < 0.001) was found to have a significant effect on depression, indicating that nonjudging of experience moderated the relationship between rumination and depression. If the interaction effect is statistically significant, a moderating effect occurs even if the effect of nonjudging experience on depression is not significant (A. F. Hayes & Rockwood, 2020; Lee, 2020).

Rumination (B = 0.51, t = 16.32, *p* < 0.001) and acting with awareness (B = −0.35, t = −5.01, *p* < 0.001) had a significant effect on depression. Additionally, the interaction effect of the two variables (B = −0.03, t = −6.09, *p* < 0.001) had a significant effect on depression, indicating that acting with awareness moderated the relationship between rumination and depression. However, describing, observing, and nonreactivity did not moderate the relationship between rumination and depression.

To examine the moderating effect of nonjudging of experience in detail, participants were divided into a high group (Mean + 1SD) and a low group (Mean − 1SD). Groups with low nonjudging of experience (B = 0.70, t = 17.22, *p* < 0.001) and low acting with awareness (B = 0.67, t = 16.35, *p* < 0.001) exhibited stronger positive relationships between rumination and depression than the respective high groups (B = 0.43, t = 10.11, *p* < 0.001; B = 0.36, t = 9.01, *p* < 0.001), supporting the partial moderating effect.

To verify the statistical significance of this interaction effect, the significance of the simple regression line between loneliness and depression was tested at specific values (Mean − 1SD, Mean, Mean + 1SD) of the moderating variable, structural social capital. All simple regression lines of loneliness on depression were significant, and the group with low structural social capital (B = 0.23, t = 14.64, *p* < 0.001) showed a stronger positive relationship between loneliness and depression than the group with high structural social capital (B = 0.17, t = 8.42, *p* < 0.001).

### 3.4. Mediated Moderating Effect

#### 3.4.1. Mediated Moderating Effect of Nonjudging of Experience on the Relationship Between Evaluative Concerns Perfectionism, Rumination, and Depression

The results are summarized in Table 4. Nonjudging of experience significantly moderates the relationship between evaluative concerns perfectionism and depression (B = −0.02, t = −4.26, *p* < 0.001). Evaluative concerns perfectionism had a significant main effect on rumination (B = 0.35, t = 11.57, *p* < 0.001). In addition, nonjudging of experience significantly moderates the relationship between rumination and depression (B = −0.02, t = −2.89, *p* < 0.01).

To verify the mediated moderating effect, the interaction term between evaluative concerns perfectionism and nonjudging of experience was examined, and the direct moderating effect remained significant (B = −0.01, t = −2.95, *p* < 0.01), indicating a partially mediated moderating effect. Bootstrapping results showed that as the level of nonjudging of experience increased, the indirect effect of the mediated moderating model decreased (Mean − 1SD = 0.25, Mean = 0.21, Mean + 1SD = 0.17).

#### 3.4.2. Mediated Moderating Effect of Acting with Awareness on the Relationship Between Evaluative Concerns Perfectionism, Rumination, and Depression

The results are summarized in Table 5. Evaluative concerns perfectionism had a significant main effect on rumination (B = 0.35, t = 11.57, *p* < 0.001). Acting with awareness significantly moderates the relationship between rumination and depression (B = −0.02, t = −2.89, *p* < 0.01).

Examination of the interaction term between evaluative concerns perfectionism and acting with awareness revealed that the direct moderating effect remained significant (B = −0.01, t = −2.72, *p* < 0.01), indicating a partially mediated moderating effect. Bootstrapping results showed that as the level of acting with awareness increased, the indirect effect of the mediated moderating model decreased (Mean − 1SD = 0.24, Mean = 0.19, Mean + 1SD = 0.15).

## 4. Discussion

This study aimed to verify the mediated moderating effect of mindfulness through rumination on the relationship between evaluative concerns perfectionism and depression in early adulthood. A comprehensive discussion of the results of this study follows.

First, evaluative concerns perfectionism was positively associated with depression, which is consistent with previous studies (Jang & Lee, 2011; Kwak & Chung, 2022). Individuals with a high degree of evaluative concerns perfectionism tend to show self-punishment, a lack of self-reinforcement, and negative self-awareness, and these characteristics are related to higher levels of depressive symptoms (M. Choi et al., 2005).

Second, rumination partially mediates the relationship between evaluative concerns perfectionism and depression. These results are consistent with previous studies reporting that evaluative concerns perfectionism is associated with rumination and, in turn, correlates with depression (Harris et al., 2008; N. H. Kim, 2016). Individuals with maladaptive perfectionism hold rigid beliefs about being perfect, focus on discrepancies between their ideal and actual self, and tend to ruminate about perceived failures (Flett et al., 2002). Notably, the mediation was partial rather than complete, as the direct association between evaluative concerns perfectionism and depressive symptoms remained statistically significant. This partial mediation indicates that rumination accounts for only a portion of the pathway linking evaluative concerns perfectionism to depressive symptoms, while the direct effect of evaluative concerns perfectionism on depression remains substantial. These findings suggest that evaluative concerns perfectionism exerts a strong direct influence on depressive symptoms, while also indicating that factors other than rumination may contribute to part of the pathway, consistent with prior studies reporting both complete and partial mediation effects.

Third, the moderating effects of nonjudging of experience and acting with awareness, which are subfactors of mindfulness, were statistically significant in the associations between perfectionism and depression. Individuals with higher levels of maladaptive perfectionism tended to report more stress and difficulties in emotional regulation, whereas higher levels of mindfulness were associated with lower perceived stress and fewer depressive tendencies, consistent with prior studies (Prakash et al., 2015). In particular, prior research has shown that nonjudging of experience and acting with awareness are associated with lower levels of depression, anxiety, and stress-related symptoms (Cash & Whittingham, 2010; Short & Mazmanian, 2013).

Fourth, nonjudging of experience and acting with awareness also moderated the association between rumination and depression. These results align with previous findings indicating that individuals with higher levels of these mindfulness facets tend to rely less on maladaptive emotion regulation strategies, such as rumination or experiential avoidance (Jury & Jose, 2019).

Finally, after examining the mediated moderating effect, nonjudging of experience and acting with awareness were identified as moderators in the model linking evaluative concerns perfectionism, rumination, and depression. These findings suggest that focusing on specific mindfulness facets may be associated with lower levels of depressive tendencies among individuals with significant evaluative concerns, although causal conclusions cannot be drawn due to the correlational design of this study.

In this study, observing, describing, and nonreactivity, which are subfactors of mindfulness, did not show significant moderating effects in any of the pathways. The lack of moderating effect of observing should be interpreted in conjunction with the nonjudging of the experience factor. In a study on college students with little meditation experience, observing was negatively correlated with nonjudging of experience, suggesting that closer attention to experiences may be linked with a lower tendency to accept experiences without judgment (Baer et al., 2004). The describing factor involves assigning words to inner experiences and emotions. Prior research argues that the describing factor may be less critical than simply being aware of inner experiences, indicating that verbal labeling alone might be insufficient to function as a protective factor in the relationship between evaluative concerns perfectionism, rumination, and depression (Josefsson et al., 2011). The nonreactivity factor primarily relates to reductions in addictive behaviors rather than depression (Eisenlohr-Moul et al., 2012). Moreover, recent findings indicate that when perseverative cognition mediates the relationship between maladaptive perfectionism and somatization, nonjudgment of experience and acting with awareness show stronger moderating associations than nonreactivity (Sun, 2022).

The contributions and clinical implications of this study are as follows. First, this study confirmed the associations of rumination and mindfulness with evaluative concerns perfectionism and depressive symptoms and, by examining their joint roles, highlighted the presence of a mediated moderating pattern. These findings indicate potential targets for mindfulness-informed interventions, though the correlational nature of the study limits causal interpretation. Second, this study examined mindfulness as a multidimensional construct and identified nonjudging of experience and acting with awareness as the only facets showing significant moderation across the examined paths. These results suggest that interventions may benefit from emphasizing these specific facets when addressing depressive tendencies related to evaluative concerns perfectionism (Baer et al., 2004). This aligns with the rationale for Acceptance and Commitment Therapy (ACT) and mindfulness-based interventions, which emphasize reducing self-criticism and enhancing present-moment awareness (S. C. Hayes et al., 2016). In particular, these facets of mindfulness can be culturally adapted to enhance clinical applicability in non-Western cultural contexts (Saggino et al., 2017).

The limitations of this study and suggestions for follow-up studies are as follows. First, this study verified the relationship between evaluative concerns perfectionism and depression based on previous studies, but it did not verify the relationship with personal standards perfectionism, which is contrary to the broader conceptualization of perfectionism. Therefore, in follow-up studies, it is necessary to explore the cognitive mechanisms that lead to depression through personal standards perfectionism and what appropriate protective factors are. Such research could provide concrete evidence for therapeutic interventions targeting personal standards perfectionism. Second, as this study was conducted using a cross-sectional design that measured only one time point, it had limitations in identifying causal relationships between variables. Without experimental manipulation, causal interpretations are limited, and even methods such as Granger causality analyses may not fully resolve this limitation. Therefore, in future studies, it is necessary to reverify this research model with appropriate time differences through longitudinal or experimental designs to strengthen causal inference. Altogether, I would like to thank the authors for their effort, but more work is required prior to accepting this manuscript for publication. I believe this study may provide a relevant contribution to the scientific literature after introducing substantial changes.

## Figures and Tables

**Table 1:** Descriptive Statistics and Correlations of Main Variables.

Variable	1	2	3	4	M	SD
1. Evaluative concerns perfectionism	1	0.64 ***	−0.54 ***	0.61 ***	92.86	18.25
2. Rumination	0.64 ***	1	−0.48 ***	0.72 ***	48.65	13.16
3. Mindfulness	−0.54 ***	−0.48 ***	1	−0.61 ***	122.01	14.75
4. Depression	0.61 ***	0.72 ***	−0.61 ***	1	38.29	10.64

*** *p* < 0.001.

**Table 2:** The moderating effect of mindfulness.

Independent Variable	Dependent Variable: Depression						
	Unstandardized Coefficient		*t*	LLCI	ULCI	F	*R* ^2^
	*B*	*SE*					
Evaluative concerns perfectionism	0.31	0.03	11.99 ***	0.26	0.37	46.20 ***	0.41
Nonjudging of experience	−0.29	0.09	−3.29 **	−0.47	−0.12		
Evaluative concerns perfectionism × Nonjudging of experience	−0.02	0.00	−4.26 ***	−0.03	−0.01		
Evaluative concerns perfectionism	0.32	0.02	13.08 ***	0.27	0.36	44.92 ***	0.40
Describing	−0.36	0.08	−4.56 ***	−0.51	−0.20		
Evaluative concerns perfectionism × Describing	0.00	0.00	0.30	−0.00	0.01		
Evaluative concerns perfectionism	0.27	0.03	10.97 ***	0.22	0.32	57.93 ***	0.47
Acting with awareness	−0.54	0.08	−7.11 ***	−0.69	−0.39		
Evaluative concerns perfectionism × Acting with awareness	−0.02	0.00	−4.87 ***	−0.02	−0.01		
Evaluative concerns perfectionism	0.35	0.02	15.08 ***	0.31	0.40	41.56 ***	0.39
Observing	−0.12	0.08	−1.61	−0.27	0.03		
Evaluative concerns perfectionism × Observing	0.01	0.00	1.96	0.00	0.01		
Evaluative concerns perfectionism	0.33	0.02	14.27 ***	0.29	0.38	46.95 ***	0.42
Nonreactivity	−0.39	0.09	−4.33 ***	−0.57	−0.21		
Evaluative concerns perfectionism × Nonreactivity	0.01	0.00	1.93	0.00	0.01		

*N* = 405, ** *p* < 0.01; *** *p* < 0.001. LLCI, lower limit confidence interval; ULCI, upper limit confidence interval.

**Table 3:** The moderating effect of mindfulness on the relationship between rumination and depression.

Independent Variable	Dependent Variable: Depression						
	Unstandardized Coefficient		*t*	LLCI	ULCI	F	*R* ^2^
	*B*	*SE*					
Rumination	0.57	0.03	17.57 ***	0.50	0.63	82.84 ***	0.56
Nonjudging of experience	−0.08	0.08	−1.02	−0.24	0.08		
Rumination × Nonjudging of experience	−0.03	0.01	−5.05 ***	−0.04	−0.02		
Rumination	0.54	0.03	19.01 ***	0.49	0.60	84.44 ***	0.56
Describing	−0.35	0.07	−5.28 ***	−0.48	−0.22		
Rumination × Describing	0.01	0.01	1.14	−0.00	0.01		
Rumination	0.51	0.03	16.32 ***	0.45	0.58	93.73 ***	0.59
Acting with awareness	−0.35	0.07	−5.01 ***	−0.49	−0.21		
Rumination × Acting with awareness	−0.03	0.00	−6.09 ***	−0.03	−0.02		
Rumination	0.61	0.03	21.83 ***	0.55	0.66	85.70 ***	0.56
Observing	−0.32	0.07	−4.94 ***	−0.45	−0.19		
Rumination × Observing	0.01	0.00	1.83	−0.00	0.02		
Rumination	0.56	0.03	20.83 ***	0.51	0.62	90.56 ***	0.58
Nonreactivity	−0.49	0.08	−6.34 ***	−0.64	−0.34		
Rumination × Nonreactivity	0.00	0.01	0.82	−0.01	0.01		

*N* = 405, *** *p* < 0.001. LLCI, lower limit confidence interval; ULCI, upper limit confidence interval.

**Table 4:** Mediated moderating effect of nonjudging via rumination on the relationship between evaluative concerns perfectionism and depression.

Step 1	Dependent variable: Depression				
	Unstandardized coefficient		*t*	LLCI	ULCI
	*B*	*SE*			
Evaluative concerns perfectionism × Nonjudging of experience	−0.02	0.00	−4.26 ***	−0.03	−0.01
Step 2	Dependent variable: Rumination				
	Unstandardized coefficient		*t*	LLCI	ULCI
	*B*	*SE*			
Evaluative concerns perfectionism	0.35	0.03	11.57 ***	0.29	0.41
Step 3	Dependent variable: Depression				
	Unstandardized coefficient		*t*	LLCI	ULCI
	*B*	*SE*			
Evaluative concerns perfectionism	0.16	0.03	6.24 ***	0.11	0.21
Nonjudging of experience	0.03	0.08	0.38	−0.12	0.18
Evaluative concerns perfectionism × Nonjudging of experience	−0.01	0.00	−2.95 **	−0.02	−0.00
Rumination	0.47	0.04	12.93 ***	0.40	0.54
Rumination × Nonjudging of experience	−0.02	0.01	−2.89 **	−0.03	−0.01

*N* = 405, ** *p* < 0.01, *** *p* < 0.001. LLCI, lower limit confidence interval; ULCI, upper limit confidence interval.

**Table 5:** Mediated moderating effect of acting with awareness via rumination on the relationship between evaluative concerns perfectionism and depression.

Step 1	Dependent variable: Depression				
	Unstandardized coefficient		*t*	LLCI	ULCI
	*B*	*SE*			
Evaluative concerns perfectionism × Acting with awareness	−0.02	0.00	−4.87 ***	−0.02	−0.01
Step 2	Dependent variable: Rumination				
	Unstandardized coefficient		*t*	LLCI	ULCI
	*B*	*SE*			
Evaluative concerns perfectionism	0.35	0.03	11.57 ***	0.29	0.41
Step 3	Dependent variable: Depression				
	Unstandardized coefficient		*t*	LLCI	ULCI
	*B*	*SE*			
Evaluative concerns perfectionism	0.13	0.02	5.28 ***	0.08	0.18
Acting with awareness	−0.27	0.07	−3.88 ***	−0.40	−0.13
Evaluative concerns perfectionism × Acting with awareness	−0.01	0.00	−2.72 **	−0.02	−0.00
Rumination	0.43	0.04	12.19 ***	0.36	0.50
Rumination × Acting with awareness	−0.02	0.01	−2.89 **	−0.03	−0.01

*N* = 405, ** *p* < 0.01, *** *p* < 0.001. LLCI, lower limit confidence interval; ULCI, upper limit confidence interval.

## Data Availability

The datasets generated and analyzed during the current study are not publicly available due to confidentiality reasons, but are available from the corresponding author upon reasonable request.
